# Ibogaine Blocks Cue- and Drug-Induced Reinstatement of Conditioned Place Preference to Ethanol in Male Mice

**DOI:** 10.3389/fphar.2021.739012

**Published:** 2021-09-21

**Authors:** Gabrielle M. Henriques, Alexia Anjos-Santos, Isa R. S. Rodrigues, Victor Nascimento-Rocha, Henrique S. Reis, Matheus Libarino-Santos, Thaísa Barros-Santos, Thais S. Yokoyama, Natalia B. Bertagna, Cristiane A. Favoretto, Célia R. G. Moraes, Fábio C. Cruz, Paulo C. R. Barbosa, Eduardo A. V. Marinho, Alexandre J. Oliveira-Lima, Laís F. Berro

**Affiliations:** ^1^ Department of Health Sciences, Universidade Estadual De Santa Cruz, Ilhéus, Brazil; ^2^ Department of Pharmacology, Universidade Federal De São Paulo, São Paulo, Brazil; ^3^ Department of Biological Sciences, Universidade Estadual De Santa Cruz, Ilhéus, Brazil; ^4^ Phytostan Do Brasil LTDA, Brasília, Brazil; ^5^ Department of Psychiatry and Human Behavior, University of Mississippi Medical Center, Jackson, MS, United States

**Keywords:** ibogaine, ethanol, conditioned place preference, mice, reinstatement

## Abstract

Ibogaine is a psychedelic extracted from the plant *Tabernanthe iboga Baill. (Apocynaceae)*, natural from Africa, and has been proposed as a potential treatment for substance use disorders. In animal models, ibogaine reduces ethanol self-administration. However, no study to date has investigated the effects of ibogaine on ethanol-induced conditioned place preference (CPP). The present study aimed to investigate the effects of repeated treatment with ibogaine on the reinstatement of CPP to ethanol in male mice. The rewarding effects of ethanol (1.8 g/kg, i. p.) or ibogaine (10 or 30 mg/kg, p. o.) were investigated using the CPP model. Furthermore, we evaluated the effects of repeated treatment with ibogaine (10 or 30 mg/kg, p. o.) on the reinstatement of ethanol-induced CPP. Reinstatement was evaluated under two conditions: 1) during a priming injection re-exposure test in which animals received a priming injection of ethanol and had free access to the CPP apparatus; 2) during a drug-free test conducted 24 h after a context-paired re-exposure, in which subjects received an injection of ethanol and were confined to the compartment previously conditioned to ethanol. Our results show that ethanol, but not ibogaine, induced CPP in mice. Treatment with ibogaine after conditioning with ethanol blocked the reinstatement of ethanol-induced CPP, both during a drug priming reinstatement test and during a drug-free test conducted after re-exposure to ethanol in the ethanol-paired compartment. Our findings add to the literature suggesting that psychedelics, in particular ibogaine, may have therapeutic properties for the treatment of alcohol use disorder at doses that do not have rewarding effects *per se*.

## Introduction

Alcohol (ethanol) use disorder (AUD) is a global public health problem and a leading cause of absenteeism and death worldwide (SAMHSA, 2018; SAMHSA, 2019; Global Status Report on Alcohol and Health, 2018). While treatment options exist, the currently available pharmacotherapies for AUD are not always effective (Fuller et al., 1986; Mason et al., 2006), and less than 20% of individuals with lifetime prevalence of AUD have ever sought treatment (Grant et al., 2015). Therefore, research on new potential treatment strategies remains a priority.

Psychedelics have long been proposed as a treatment for drug abuse, including AUD (Bogenschutz and Johnson, 2016). A meta-analysis of randomized controlled trials administering a single high-dose of lysergic acid diethylamide (LSD) for the treatment of AUD showed that 59% of LSD patients improved at initial follow-up compared to 38% of control patients (Krebs and Johansen, 2012). Similarly, in a study investigating psychedelic-assisted treatment for AUD, acute treatment with psilocybin significantly decreased drinking days and heavy drinking days for 32 weeks compared to baseline (Bogenschutz et al., 2015). Studies from our research group also have shown that ayahuasca, a hallucinogenic substance, blocks the development and expression of ethanol-induced behavioral sensitization (Oliveira-Lima et al., 2015) and the expression of conditioned place preference (CPP) to ethanol (Cata-Preta et al., 2018) in mice.

Ibogaine (IBO) is another psychedelic extract that has been proposed as a potential treatment for substance use disorder (SUD) (Brown, 2013). IBO is extracted from the plant *Tabernanthe iboga Baill. (Apocynaceae)*, natural from Africa (Heink et al., 2017; Corkery, 2018), and is commonly consumed during religious ceremonies in the form of a tea made from the plant’s stem and root bark (Goutarel et al., 1993). The discovery of its potential for the treatment of SUD occurred in the 1980s by Howard Lotsof, when he first proposed the therapeutic use of IBO for treating heroin abuse (Lotsof, 1985) and AUD (Lotsof, 1989).

Particularly for ethanol, studies have shown that treatment with IBO reduced ethanol self-administration in rats (Lotsof, 1989; Rezvani et al., 1995; He et al., 2005). A more recent study investigating retrospective data from SUD patients (with 14% or participants indicating ethanol abuse) who used IBO in the past showed significant improvements in withdrawal and cravings following IBO use (Heink et al., 2017). However, to the best of our knowledge no study to date has investigated the effects of IBO on ethanol-induced CPP. Of note, while IBO also has been shown to reduce morphine self-administration (Glick et al., 1991), it only blocked the development, but not the expression, of morphine-induced CPP (Luxton et al., 1996; Parker and Siegel, 2001), emphasizing the importance of investigating the effects of IBO on several abuse-related measures.

The present study aimed to investigate the effects of repeated IBO treatment on the reinstatement of ethanol-induced CPP under two conditions: 1) during a priming injection re-exposure test in which animals received a priming injection of ethanol and had free access to the CPP apparatus; 2) during a drug-free test conducted 24 h after a context-paired re-exposure, in which subjects received an injection of ethanol and were confined to the compartment previously conditioned to ethanol. Given our previous findings showing rewarding effects of another psychedelic substance (Cata-Preta et al., 2018; Reis et al., 2020), we also investigated whether IBO induced CPP in mice.

## Materials and Methods

### Animals

Three-month-old Swiss male mice from our own colony were used. Animals weighing 35–40 g were group housed (8 per cage) in polypropylene cages (41 × 34 × 16.5 cm) under controlled temperature (22–23°C) and light (12 h light, 12 h dark; lights on at 6:45am) conditions. Rodent chow (Nuvilab, Quimtia SA, Colombo, PR, Brazil) and water were available ad libitum throughout the experiments. Animals were maintained according to the National Institutes of Health Guide for the Care and Use of Laboratory Animals (8th Edition, revised 2011) and in accordance with the Brazilian Law for Procedures for Animal Scientific Use (#11794/2008). The Institutional Animal Care and Use Committee of UESC approved the experimental procedure (protocol #006/2017).

### Drugs

Absolute ethanol (Merck®) was diluted in distilled water to the dose of 1.8 g/kg and administered intraperitoneally (i.p.) at a volume of 10 ml/kg of body weight. Ibogaíne (IBO) was obtained in crystal form (12- Methoxybogainamide, Biogen®) and diluted in distilled water +50 µL of tween 10 which was used as vehicle (Veh) solution. IBO e Veh solutions were administrated orally (gavage). The dose of ethanol was chosen based on previous studies in our laboratory using the CPP paradigm (Silva et al., 2017; Cata-Preta et al., 2018; Libarino-Santos et al., 2020). The doses of IBO were chosen based on previous rodent studies investigating its effects on ethanol self-administration (Rezvani et al., 1995; He et al., 2005).

### Conditioned Place Preference

The CPP apparatus consisted of two conditioning compartments of equal size (40 × 20 × 20 cm): compartment A, with black and white vertical lines on the walls and a black wooden floor, and compartment B, with black and white horizontal lines on the walls and a dark (red) smooth floor, both connected by a central choice compartment (40 × 10 × 15 cm) that was accessible by sliding doors. During test sessions, the time spent in each compartment was registered using the ANY-maze software (version 5.1, Stoelting) and a webcam suspended overhead. Expression of drug-induced CPP was evidenced by the CPP score (time spent in the drug-paired compartment minus time spent in the saline-paired compartment). For animals who received saline in both compartments (SAL-SAL group), the CPP score was established by randomly assigning a reference compartment (e.g., time spent in compartment A minus time spent in compartment B, or vice versa). Total distance traveled in the CPP apparatus also was measured during tests. The CPP design including a treatment phase, post-treatment test, alcohol re-exposure and post-re-exposure (reinstatement) test has been used in our laboratory for several years, with reliable results (Silva et al., 2017; Cata-Preta et al., 2018; Reis et al., 2020; Libarino-Santos et al., 2020; Libarino-Santos et al., 2021). This protocol (including the choice of ethanol dose) has been used in our laboratory for several years, and has been shown to reliably induce CPP in mice, allow enough time for extinction and be sensitive to reinstatement upon an ethanol re-exposure, while allowing for treatment manipulations during extinction. The CPP procedure consisted of the following phases:

*Habituation (Days 1–2):* For 2 consecutive days, animals were placed in the center of the apparatus with the door open with free access to both compartments for 15 min. No injection was administered.

*Pre-conditioning test (Day 3):* Animals were placed in the center of the apparatus with the door open with free access to both compartments and behavior was recorded for 15 min. No treatments were administered on the day of the pre-conditioning test.

*Conditioning (Days 4–11):* An unbiased design was used because animals showed no preference for either of the compartments in the pre-conditioning test. Therefore, animals were randomly assigned to an experimental group and to an “ethanol-paired compartment” in a counterbalanced manner. The conditioning sessions were performed during 8 consecutive days, during which the doors remained closed and animals were confined to one of the conditioning compartments. Animals received an administration of saline on odd days and drug (Experiment 1: ethanol or IBO; Experiment 2: ethanol) on even days. Five minutes after saline or ethanol injections, or 30 min after IBO injections, mice were confined to the assigned drug- or Sal-paired compartment for 10 min.

*Post-conditioning test (Day 12):* Animals were placed in the center of the apparatus with the door open with free access to both compartments and behavior was recorded for 15 min. No treatments were administered on the day of the post-conditioning test.

*Treatment (Days 13–20):* For 8 consecutive days, animals received daily oral administrations of IBO or vehicle (Veh) on odd days, and saline on even days. Thirty min after injections, animals were confined to the assigned ethanol- (IBO or Veh treatments) or saline- (saline treatments) paired compartments for 10 min.

*Post-treatment test (Day 21):* Animals were placed in the center of the apparatus with the door open with free access to both compartments and behavior was recorded for 15 min. No treatments were administered on the day of the post-treatment test.

*Priming injection re-exposure test (Day 22):* Twenty-four hours after the post-treatment test, half of the animals in Experiment 2 received an i. p. injection of ethanol (1.8 g/kg) and, 5 min after injection, were placed in the center of the apparatus with the door open with free access to both compartments and behavior was recorded for 15 min.

*Context-paired re-exposure (Day 22):* Twenty-four hours after the post-treatment test, half of the animals in Experiment 2 received an i. p. injection of ethanol (1.8 g/kg) and, 5 min after injection, were confined to the ethanol-paired compartment for 10 min.

*Post-context-paired re-exposure test (Day 23)*: Animals that were submitted to the context-paired re-exposure were placed in the center of the apparatus with the door open with free access to both compartments and behavior was recorded for 15 min. No treatments were administered on the day of the post-context-paired re-exposure test.

All behavioral sessions were conducted during the same period of the day within an experiment. Because several groups were running concomitantly within a given experiment, the order of animals being submitted to the behavioral sessions in the CPP was randomized for each phase described above, so that all groups had animals being tested at the same time. The CPP apparatus was cleaned with ethanol-water (5%) solution before each behavioral session/test to eliminate possible bias due to odors left by previous mice. Different cohorts of mice were used for each experiment described below.

### Experimental Design

**FIGURE 1:**
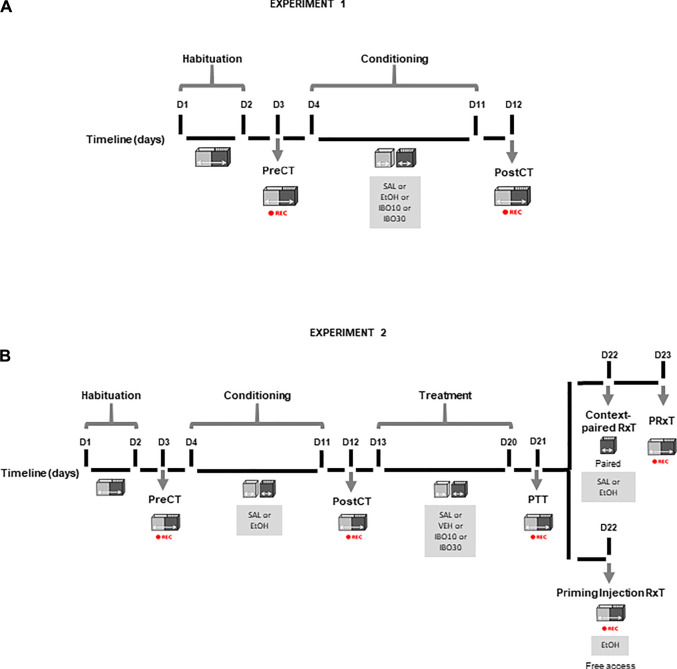
Conditioned place preference (CPP) protocol design across experimental days (D). **(A)** Experiment 1 **(B)** Experiment 2. PreCT: drug-free pre-conditioning test. Conditioning: saline (SAL), ethanol (EtOH, 1.8 g/kg) or ibogaine (IBO, 10 or 30 mg/kg) conditioning alternated with saline conditioning. PostCT: drug-free post-conditioning test. Treatment: saline (SAL), vehicle (VEH) or ibogaine (IBO, 10 or 30 mg/kg) treatment in the compartment previously paired with ethanol; PTT: drug-free post-treatment test. Context-paired RxT: context-paired re-exposure, in which animals received an injection of saline (SAL) or ethanol (EtOH, 1.8 g/kg) and were confined to the compartment previously paired with ethanol. PRxT: drug-free post-context-paired re-exposure test. Priming Injection RxT: priming injection re-exposure test, in which animals received an injection of ethanol (EtOH, 1.8 g/kg) and had free access to both compartments of the CPP apparatus. REC: session recording for analysis using the AnyMaze software.

The experimental design for Experiments 1 and 2 is illustrated in Figure 1.

#### Experiment 1: Effects of Treatment With Ibogaine or Ethanol on the CPP Paradigm

In order to evaluate whether IBO or ethanol would induce CPP, mice were submitted to the habituation, pre-conditioning test and IBO (10 or 30 mg/kg, *n* = 8 per group, IBO10 and IBO30 groups) or ethanol (1.8 g/kg, *n* = 8, EtOH group) conditioning followed by post-conditioning test as previously described. A control group (*n* = 8, SAL group) that underwent habituation, pre-conditioning treatment with saline on both compartments during the conditioning phase, and a post-conditioning test also was included.

#### Experiment 2: Effects of Treatment With IBO on Drug- or Cue-Induced Reinstatement of Ethanol-Induced Conditioned Place Preference

Seventy-two mice were submitted to the habituation, pre-conditioning test, ethanol (1.8 g/kg) conditioning and post-conditioning test as previously described. A control group (*n* = 24) underwent habituation, pre-conditioning treatment with saline on both compartments during the conditioning phase, and a post-conditioning test (SAL-SAL group).

Twenty-four hours after the post-conditioning test, the treatment phase began. For 8 days, animals received an oral administration of either Veh (ETH-VEH group, *n* = 24) or IBO at the doses of 10 mg/kg (ETH-IBO10 group, *n* = 24) or 30 mg/kg (ETH-IBO30 group, *n* = 24) every other day on even days and, 30 min after injection, were confined to the compartment previously paired with ethanol for 10 min. On odd days, animals received an oral administration of saline associated with the opposite (saline-paired) compartment. A control group (SAL-SAL, *n* = 24 animals) was treated with saline on both compartments.

The treatment phase was followed by the drug free post-treatment test. On the following day, a subgroup of animals (*n* = 16 per group) was submitted to the priming injection re-exposure test. All animals that were submitted to this phase (including the SAL-SAL group) received an i. p. injection of ethanol (1.8 g/kg).

The remaining animals (*n* = 8 per group) were submitted to the context-paired re-exposure, during which all animals previously conditioned with ethanol received an ethanol injection (1.8 g/kg) and were confined to the ethanol-paired compartment. Animals in the SAL-SAL group received a saline injection and were randomly confined to one of the two saline-paired compartments. This phase was followed by the drug free post-context-paired re-exposure test.

### Statistical Analysis

All variables were checked for normality (Shapiro–Wilk test) and homogeneity of variances (Levene’s test), which validated the use of parametric tests. Multiple comparisons were performed using one-way or two-way analysis of variance (ANOVA). When two-way repeated measures (RM) ANOVA was used, two variables were analyzed: Treatment (ethanol vs saline) and Phases (pre-vs post-conditioning). When appropriate, Bonferroni’s post-hoc test was then performed for multiple comparisons between groups. In all comparisons, a *p* value below 0.05 was considered a statistically significant effect.

## Results

### Experiment 1: Effects of Treatment With Ibogaine or Ethanol on the Conditioned Place Preference Paradigm

#### Conditioned Place Preference Score

**FIGURE 2:**
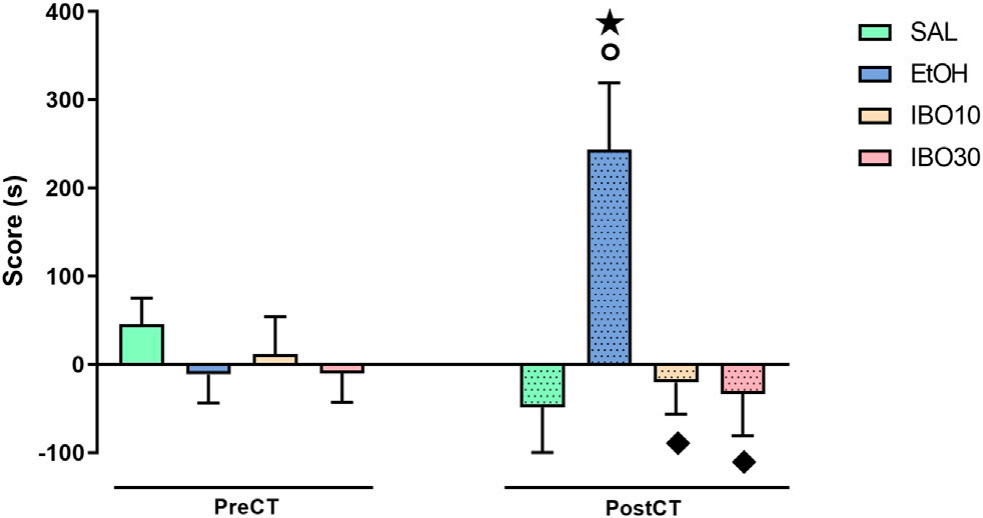
Effects of conditioning with saline (SAL, i. p.), ethanol (EtOH, 1.8 g/kg, i. p.) or ibogaine (IBO, 10 or 30 mg/kg, oral administration) in the conditioned place preference (CPP) paradigm. CPP score (difference between the time spent in the EtOH/IBO- and in the saline-paired compartments) during the pre-conditioning test (preCT, *n* = 8 per group) and the post-conditioning test (postCT, *n* = 8 per group) sessions. Data are reported as means ± SEM. **★** *p* < 0.05 compared with the same group in the PreCT; °*p* < 0.05 compared with the SAL group in the PostCT; *p* < 0.05 compared with the EtOH group in the PostCT.

Two-way RM ANOVA showed a significant interaction between treatment (saline vs ethanol vs IBO) and time (pre-conditioning test vs post-conditioning test) for CPP score [F (3,28) = 6.564; *p* = 0.0017] (Figure 2). Bonferroni post-hoc test showed that animals conditioned with ethanol showed a significant increase in CPP score compared to the same group during the pre-conditioning test (*p* = 0.0061) and to the SAL group in the post-conditioning test (*p* = 0.0009). Animals conditioned with IBO, on the other hand, did not significantly differ from themselves in the pre-conditioning test or from the SAL group in the post-conditioning test, and showed a lower CPP score compared the EtOH group during the post-conditioning test (IBO10: *p* = 0.0038; IBO30: *p* = 0.0019).

#### Distance Travelled

**FIGURE 3:**
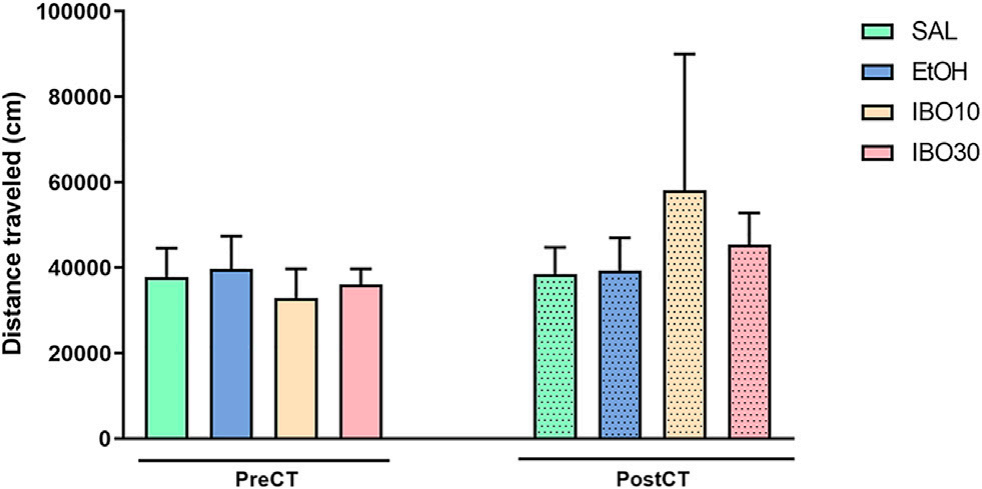
Effects of conditioning with saline (SAL, i. p.), ethanol (EtOH, 1.8 g/kg, i. p.) or ibogaine (IBO, 10 or 30 mg/kg, oral administration) in the conditioned place preference (CPP) paradigm. Total distance traveled in the CPP apparatus during the pre-conditioning test (preCT, *n* = 8 per group) and the post-conditioning test (postCT, *n* = 8 per group) sessions. Data are reported as means ± SEM.

Two-way ANOVA showed no significant effects of time [F (1, 28) = 1.023; *p* = 0.3206], treatment [F (3, 28) = 0.1134; *p* = 0.9515], or interaction [F (3, 28) = 0.4651; *p* = 0.7090] in the total distance travelled in the CPP apparatus during the pre- and post-conditioning tests (Figure 3).

### Experiment 2: Effects of treatment with Ibogaine on drug- or cue-induced reinstatement of ethanol-induced Conditioned Place Preference

#### Conditioned Place Preference Score

**FIGURE 4:**
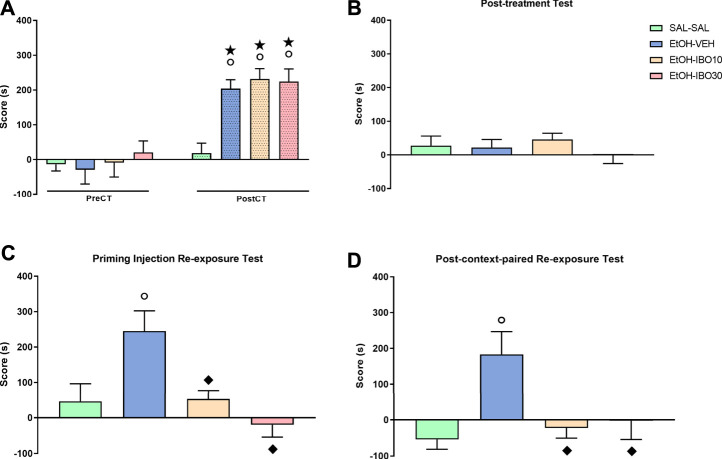
Effects of treatment with ibogaine on the reinstatement of ethanol-induced conditioned place preference (CPP). **(A)** CPP score (difference between the time spent in the ethanol- and in the saline-paired compartments) during the pre-conditioning test (preCT, *n* = 24 per group) and the post-conditioning test (postCT, *n* = 24 per group) following conditioning with saline (SAL, i. p.) or ethanol (EtOH, 1.8 g/kg, i. p.) in the CPP apparatus. **(B)** CPP score during a drug-free post-treatment test (*n* = 24 per group) after treatment with saline (SAL, i. p.), vehicle (VEH, oral administration) or ibogaine (IBO, 10 or 30 mg/kg, oral administration) in the ethanol-paired compartment. **(C)** CPP score during a priming injection re-exposure test in which all groups received a priming injection of ethanol (EtOH, 1.8 g/kg, i. p.) and had free access to the CPP apparatus (*n* = 8 per group). **(D)** CPP score during a drug-free test conducted 24 h after a context-paired re-exposure, in which subjects received an injection of saline (SAL-SAL group, *n* = 16) or ethanol (EtOH, 1.8 g/kg, remaining groups, *n* = 16 per group) and were confined to the compartment previously conditioned to ethanol. Data are reported as means ± SEM. **★** *p* < 0.05 compared with the same group in the PreCT; °*p* < 0.05 compared with the SAL group in the same experimental phase; *p* < 0.05 compared with the EtOH group in the same experimental phase.

Two-way RM ANOVA of the pre- and post-conditioning tests showed a significant interaction between treatment (saline vs ethanol vs IBO) and time (pre-conditioning test vs post-conditioning test) for CPP score [F (3, 92) = 5.679; *p* = 0.0013] (Figure 4A). Bonferroni post-hoc test showed that all groups conditioned with ethanol (EtOH-VEH, EtOH-IBO10 and EtOH-IBO30) showed a significant increase in CPP score compared to the same group during the pre-conditioning test (*p* < 0.0001 for all three groups) and to the SAL group in the post-conditioning test (*p* < 0.001 for all three groups).

During the post-treatment test, one-way ANOVA showed no significant differences between groups [F (3, 92) = 0.6296; *p* = 0.5977], showing that after the treatment with Veh or IBO the animals no longer expressed preference for the ethanol-paired compartment compared to the SAL-SAL group, indicative of extinction (Figure 4B).

For the animals that were submitted to the priming injection re-exposure test, one-way ANOVA showed a significant difference between groups [F (3, 60) = 6.810; *p* = 0.0005] (Figure 4C). Ethanol re-exposure reinstated ethanol-induced CPP, with animals in the VEH-EtOH group showing an increased CPP score compared to the animals in the SAL-SAL group while under the effects of ethanol (*p* = 0.012). Treatment with IBO at the doses of 10 and 30 mg/kg blocked ethanol-induced reinstatement during the priming injection re-exposure test, with animals in the EtOH-IBO10 and EtOH-IBO30 groups not differing from the SAL-SAL group, but showing decreased CPP score compared to the VEH-EtOH group (IBO 10: *p* = 0.017; IBO30: *p* = 0.0004).

For animals submitted to the context-paired re-exposure and subsequent test, one way-ANOVA also showed a significant difference between groups during the test [F (3, 26) = 5.339; *p* = 0.0053] (Figure 4D). Exposure to ethanol and the ethanol-paired compartment the previous day induced reinstatement of ethanol-induced CPP even when animals were no longer under the drug effect, with animals in the VEH-EtOH group showing an increased CPP score compared to the animals in the SAL-SAL group (*p* = 0.0078). Treatment with IBO at the doses of 10 and 30 mg/kg blocked ethanol- and context-induced reinstatement, with animals in the EtOH-IBO10 and EtOH-IBO30 groups not differing from the SAL-SAL group, but showing decreased CPP score compared to the VEH-EtOH group (IBO 10: *p* = 0.02; IBO30: *p* = 0.04).

#### Distance Travelled

**FIGURE 5:**
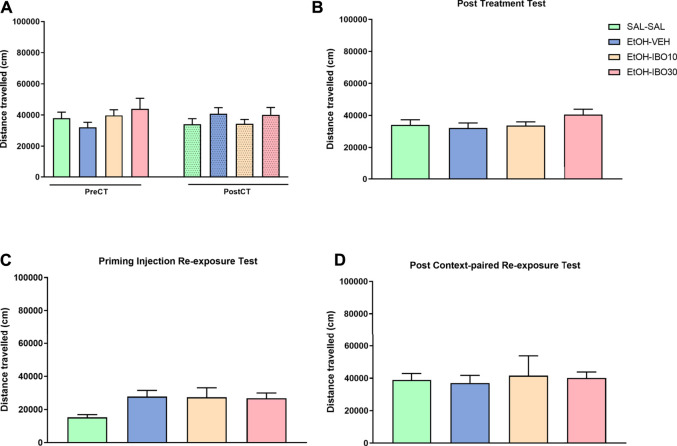
Effects of treatment with ibogaine on the reinstatement of ethanol-induced conditioned place preference (CPP). **(A)** Total distance traveled in the CPP apparatus during the pre-conditioning test (preCT, *n* = 24 per group) and the post-conditioning test (postCT, *n* = 24 per group) following conditioning with saline (SAL, i. p.) or ethanol (EtOH, 1.8 g/kg, i. p.) in the CPP apparatus. **(B)** Total distance traveled during a drug-free post-treatment test (*n* = 24 per group) after treatment with saline (SAL, i. p.), vehicle (VEH, oral administration) or ibogaine (IBO, 10 or 30 mg/kg, oral administration) in the ethanol-paired compartment. **(C)** Total distance traveled during a priming injection re-exposure test in which all groups received a priming injection of ethanol (EtOH, 1.8 g/kg, i. p.) and had free access to the CPP apparatus (*n* = 8 per group). **(D)** Total distance traveled during a drug-free test conducted 24 h after a context-paired re-exposure, in which subjects received an injection of saline (SAL-SAL group, *n* = 16) or ethanol (EtOH, 1.8 g/kg, remaining groups, *n* = 16 per group) and were confined to the compartment previously conditioned to ethanol. Data are reported as means ± SEM.

No significant effects were observed for distance traveled in the analysis of pre-vs post-conditioning (two-way RM ANOVA, time [F (1, 92) = 0.2027; *p* = 0.6536]; treatment [F (3, 92) = 0.6357; *p* = 0.5939]; interaction [F (3, 92) = 1.894; *p* = 0.1361]), post-treatment test (one-way ANOVA [F (3, 92) = 1.469; *p* = 0.2283]), priming injection re-exposure test (one-way ANOVA [F (3, 60) = 2.442; *p* = 0.0729]) or post-context-paired re-exposure test (one-way ANOVA [F (3, 26) = 0.06801; *p* = 0.9764]) (Figure 5).

## Discussion

Psychedelics have long been proposed as a treatment for drug abuse, and studies have shown that the plant extract IBO blocks some abuse-related effects of ethanol in rats and humans. The present study adds to the literature by showing for the first time that treatment with IBO blocked prime- and cue-induced reinstatement of CPP to ethanol in mice at doses that did not induce CPP *per se*.

Our findings show that treatment with IBO in the ethanol-paired compartment blocked the reinstatement of ethanol-induced CPP. Importantly, these effects were observed 24 h or more after the last IBO treatment, and IBO was effective in blocking both an ethanol-priming reinstatement (test conducted with ethanol on board) and an ethanol- and context-induced reinstatement (drug-free test conducted 24 h after an ethanol re-exposure in the drug-paired compartment). These findings are in agreement with previous studies showing that treatment with IBO and its derivatives blocked ethanol self-administration in rats (Lotsof, 1989; Rezvani et al., 1995; Rezvani et al., 2016; He et al., 2005).

Ethanol-induced increased dopamine levels in the nucleus accumbens (NAc, Imperato and Di Chiara 1986) via increased firing of dopaminergic cells in the ventral tegmental area (VTA, Brodie et al., 1990 has been proposed to mediate its rewarding effects (Wise and Rompre 1989; Kalivas, 2002). The pharmacological properties of IBO are complex, and this compound binds to several receptors that are involved in the rewarding effects of ethanol. IBO acts as a 5-HT_2_ receptor agonist and has higher affinity for 5-HT_2C_ over 5-HT_2A_ receptors (Glick et al., 2001; Corkery, 2018). Activation of 5-HT_2C_ receptors, which are highly expressed in GABAergic interneurons within the VTA, decreases the activity of VTA dopamine neurons and, consequently, NAc dopamine levels (Howell and Cunningham, 2015), which could partially explain the present findings. In fact, 5-HT_2C_ receptor agonists have been shown to block the abuse-related behavioral effects of drugs of abuse (Manvich et al., 2012; Berro et al., 2017), including ethanol self-administration (Rezvani et al., 2014; Tabbara et al., 2021).

In addition to 5-HT_2C_ receptors, IBO also acts as an agonist at κ-opioid receptors (Glick et al., 2001; Schep et al., 2016). κ-opioid receptor antagonists potentiate ethanol-induced CPP (Matsuzawa et al., 1999), while κ-opioid receptor agonists induce conditioned place aversion in rodents (Bals-kubik et al., 1993). Considering that in the present study treatment with IBO was paired with the compartment previously associated with ethanol, the κ-opioid receptor agonist activity of IBO may have altered the incentive salience to ethanol-predictive cues (Valyear et al., 2017). This effect could have decreases the motivation for ethanol-seeking behavior and, consequently, blocked reinstatement. Importantly, IBO also acts as an antagonist at N-methyl-d-aspartate (NMDA) receptors (Glick et al., 1997). Recent studies have shown that activation of NMDA receptors is a key mechanism responsible for the generation of conditioned responses of dopamine neurons to reward cues, and that blockade of NMDA receptors also disrupts attribution of incentive salience to reward-paired stimuli (Cieślak and Rodriguez Parkitna, 2019). In fact, NMDA receptor antagonists block the expression of ethanol-induced CPP in mice (Gremel and Cunningham, 2009; Gremel and Cunningham, 2010).

IBO also has been shown to facilitate memory retrieval (Popik, 1996). Therefore, we propose that treatment with IBO in the ethanol-paired compartment may have facilitated the retrieval of ethanol-associated conditioned memories. IBO’s action at 5-HT_2C_, κ-opioid and NMDA receptors would then alter the incentive salience of ethanol-associated cues, blocking a subsequent reinstatement of ethanol-induced CPP upon an ethanol re-exposure. Together, these findings suggest that IBO acts at several neurotransmitter systems involved in ethanol reward and reinstatement, and that a treatment with IBO would prevent the downstream effects of an ethanol re-exposure that would lead to reinstatement.

Our findings are in agreement with previous studies showing that IBO attenuated ethanol self-administration in rats (Lotsof, 1989; Rezvani et al., 1995; Rezvani et al., 2016; He et al., 2005) and withdrawal and craving in users of ethanol and other drugs (Heink et al., 2017). The pharmacology of IBO is complex, and its therapeutic effects may be better explained by a combined activity at different neurotransmitter systems involved in AUD. Further studies are needed to elucidate the precise receptor subtypes and mechanisms underlying the therapeutic effects of IBO on ethanol reward, reinforcement and reinstatement. Importantly, this study adds to the growing literature suggesting that IBO may be a useful therapeutic tool in the treatment of drug abuse and AUD (Brown, 2013). Evidence has pointed to an increasing number of individuals with substance use disorders seeking out treatment using psychoactive substances (MAPS 2003), emphasizing the importance of future controlled clinical trials investigating the safety and efficacy of psychoactive substances for the treatment of AUD.

## Data Availability

The raw data supporting the conclusions of this article will be made available by the authors, without undue reservation.
